# Metastatic mixed adenoneuroendocrine carcinoma of the liver successfully resected by hepatic trisectionectomy following chemotherapy: A case report

**DOI:** 10.1002/ccr3.1968

**Published:** 2019-02-01

**Authors:** Osamu Sato, Takahiro Tsuchikawa, Toru Yamada, Daisuke Sato, Yoshitsugu Nakanishi, Toshimichi Asano, Takehiro Noji, Kurashima Yo, Yuma Ebihara, Soichi Murakami, Toru Nakamura, Keisuke Okamura, Toshiaki Shichinohe, Tomoko Mitsuhashi, Satoshi Hirano

**Affiliations:** ^1^ Department of Gastroenterological Surgery II Hokkaido University Faculty of Medicine Sapporo Japan; ^2^ Department of Surgical Pathology Hokkaido University Hospital Sapporo Japan

**Keywords:** immunohistochemistry, mixed adenoneuroendocrine carcinoma, neuroendocrine carcinoma, neuroendocrine tumor

## Abstract

The chemotherapy guidelines for mixed adenoneuroendocrine carcinoma (MANEC) remain poorly defined, and prognosis remains dismal. In this case, we successfully performed resection after FOLFOX for unresectable metastatic MANEC of the liver. Thus, chemotherapy for adenocarcinoma may be effective for MANEC.

## 1 INTRODUCTION

Mixed adenoneuroendocrine carcinomas (MANECs) are rare malignancies that have poor prognoses because of their aggressive characteristics such as rapid growth and early vascular invasion, leading to system‐wide dissemination.^1^, ^2^ According to the 2010 World Health Organization (WHO) classification, MANECs are characterized by both a neuroendocrine and an adenocarcinomatous component, each of which accounts for at least 30% of the lesion.^3^ Moreover, such tumors, which are frequently unresectable, usually require multidisciplinary therapy or (if resected) postoperative adjuvant therapy. However, the most effective chemotherapy regimen for patients with MANEC remains unclear. Herein, we report about a patient with colorectal MANEC and liver metastases who successfully underwent resection of these metastatic lesions after conversion chemotherapy and obtained a survival benefit.

## 2 CASE PRESENTATION

**Figure 1:**
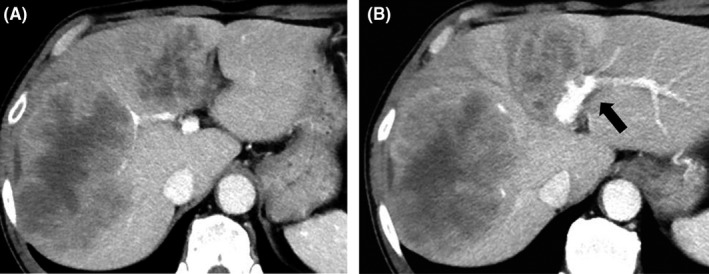
Initial enhanced computed tomography images. A, Multiple low‐density tumors with enhanced margins in the liver. B, The tumors invading the umbilical portion (arrow)

**Figure 2:**
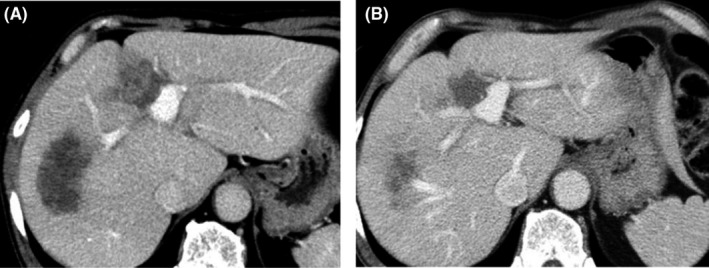
Enhanced computed tomography images after adjuvant chemotherapy. A, Images after eight rounds of adjuvant chemotherapy showing that the liver tumors decreased in size. B, Images after additional chemotherapy showing further reduction in the sizes of the liver tumors and improvement of the invaded umbilical portion

A 62‐year‐old man underwent sigmoidectomy after being diagnosed with sigmoid colon cancer and multiple metastases in the liver (S3, S4, S6, and S8) at a nearby hospital. The histopathological diagnosis of the sigmoidectomy specimen was adenocarcinoma, type 2, por1>tub2, pT3N1M1a(H2), pStage IV according to the TNM classification. The tumor had a wild‐type KRAS status. The liver metastases were deemed unresectable because they invaded the umbilical portion; therefore, the patient underwent eight cycles of adjuvant chemotherapy with 5‐fluorouracil and oxaliplatin (FOLFOX) + panitumumab. Follow‐up abdominal computed tomography after completing chemotherapy showed remarkable shrinkage of the liver tumors; a partial response was observed, that is, a 44% decrease in the sum of target lesions according to the revised Response Evaluation Criteria in Solid Tumors, version 1.1, guidelines. Afterward, the patient was scheduled to undergo five cycles of the same chemotherapy for further shrinkage of the tumor; however, because panitumumab‐induced adverse effects appeared, three of these cycles comprising FOLFOX only were administered. Following these chemotherapy cycles, the patient had stable disease, with only slight reductions in tumor sizes, that is, a stable disease with a 10% decrease in the sum of target lesions (Figures 1 and 2). The lesions were deemed resectable at this point, that is, 15 months after the initial sigmoidectomy. The patient underwent left hepatic trisectionectomy and partial posterior segmentectomy accompanied by partial right hepatic vein resection three weeks after treatment with percutaneous transhepatic portal vein embolization therapy at the left and anterior segment branches of the portal vein. The surgery lasted for 630 minutes, and the intraoperative hemorrhage volume was 3540 mL.

**Figure 3:**
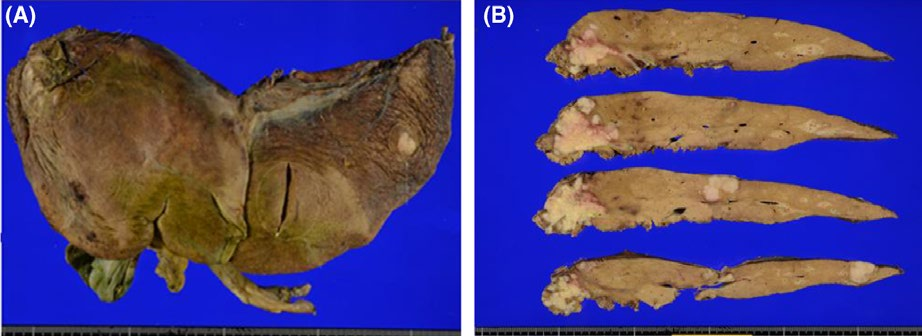
Gross findings of the trisectionectomy specimen of the liver. A, The outer surface of the specimen. B, Cut surfaces of the cross‐sectioned specimen

Gross examination of the hepatic lesion showed that all tumors exhibited a confluent multinodular growth. Cross‐sections of the tumor showed white to yellowish‐white, with varying edematous and sclerotic background (Figure 3). Histopathological examination revealed that most of the tumors had relatively uniform oval nuclei and eosinophilic cytoplasm. In most areas of each lesion, the tumors showed an organoid growth pattern without forming conspicuous glandular lumens. These histopathological findings were not typical of colon cancers.

**Figure 4:**
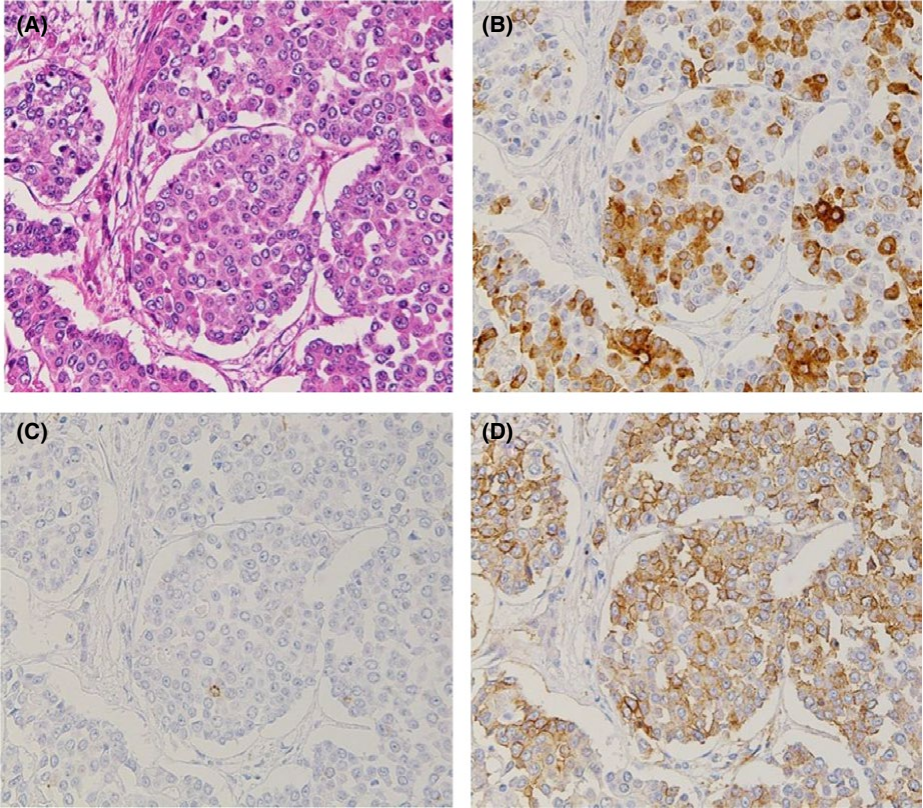
Microscopic findings of the surgically resected liver tumor (magnification, ×200). A, Hematoxylin and eosin staining showing that the tumor mainly had an organoid pattern. B, Immunohistochemical staining of synaptophysin. C, Immunohistochemical staining of chromogranin A. D, Immunohistochemical staining of CD56

**Figure 5:**
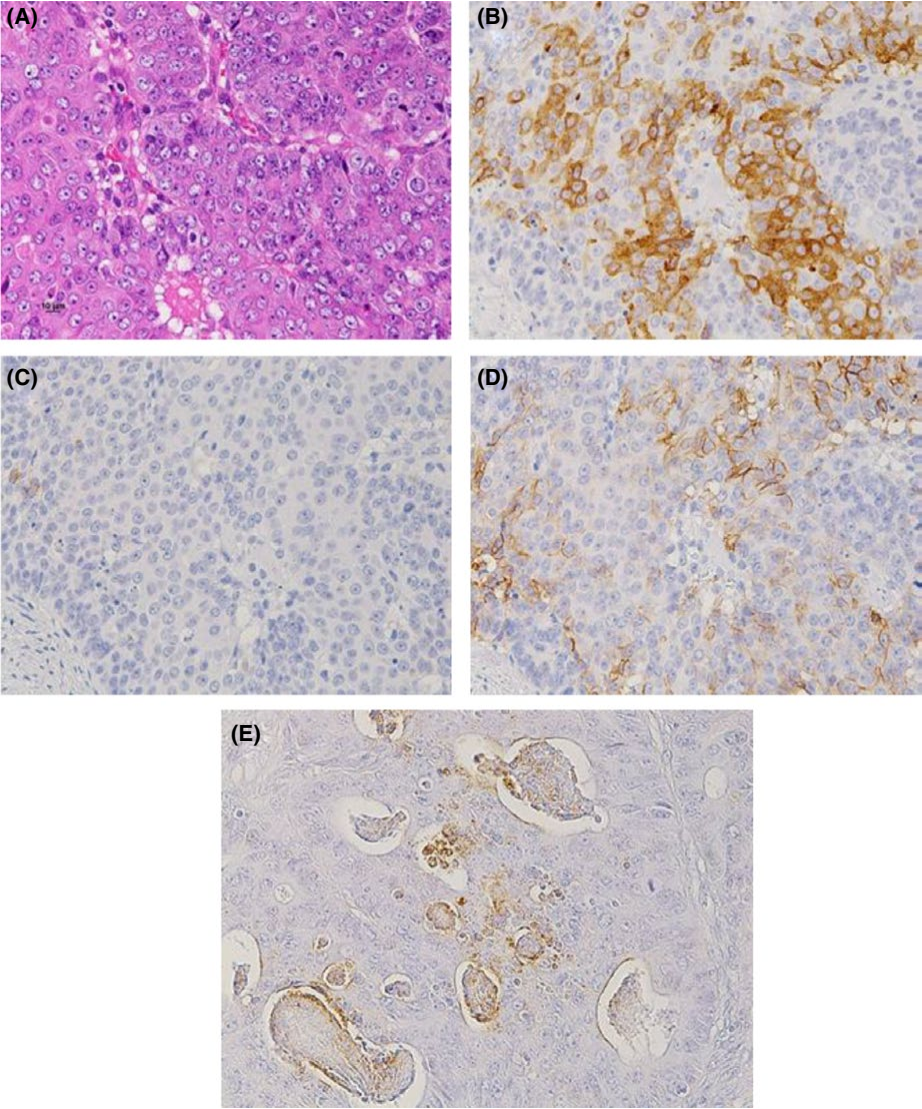
Microscopic findings of the surgically resected primary tumor (magnification, ×200). A, Hematoxylin and eosin staining showing the area of a compact alveolar pattern without conspicuous glandular lumens. B, Immunohistochemical staining of CD56. C, Immunohistochemical staining of chromogranin A. D, Immunohistochemical staining of synaptophysin. E, Immunohistochemical staining of carcinoembryonic antigen in the areas with glandular formation

Immunohistochemical examination revealed positive stainings for synaptophysin and CD56 in most areas of each lesion, and chromogranin A was also weakly positive (Figure 4). The morphological and immunohistochemical findings of these tumors resembled neuroendocrine carcinoma (NEC), which was considered among the differential diagnoses of MANEC. To make a definitive diagnosis, we assessed the primary tumor of the sigmoid colon and found the areas consistent with NEC. The morphological and immunohistochemical patterns of the primary tumor were similar to those of the liver tumors, and the Ki‐67 labeling index was 57.7%. In addition, there were dominantly adenocarcinomatous components that formed glandular lumens with immunohistochemically positive staining for carcinoembryonic antigen (CEA) (Figure 5). Furthermore, because these components, each of a NEC and an adenocarcinoma, accounted for ≥30% of the areas of each lesion, the final histopathological diagnosis was MANEC of the colon with multiple liver metastases. In some areas, CEA‐positive cells were partly overlapped with NET marker‐positive cells, so we considered that the primary tumor was an amphicrine tumor rather than a collision tumor. Although there were no conspicuous glandular lumens in the liver tumors, we confirmed positive staining for CEA in the liver tumors and in the areas forming the glandular lumens by immunohistochemistry. The histological effect of chemotherapy was equivalent to grade 2, according to the Evans classification system.^5^

The patient commenced oral capecitabine administration after surgery, but recurrences of the liver metastases were observed in the caudate lobe and retroperitoneum. The patient died of cancer 17 months after the liver resection, that is, 35 months after the initial sigmoidectomy.

## 3 DISCUSSION

Neuroendocrine components are frequently observed in gastrointestinal adenomas/adenocarcinomas in routine clinical practice. However, mixed exocrine‐neuroendocrine tumors are rarely observed.^1^ According to the WHO classification in 2010, neuroendocrine tumors (NETs) can be divided into three categories based on the mitotic count and Ki‐67 labeling index: G1, G2, and NEC. Furthermore, MANECs are defined as tumors comprising at least 30% of a neuroendocrine and an adenocarcinomatous component.^2^ MANECs have been reported to be both rapidly progressive and highly proliferative, and the prognosis of patients with these tumors is poorer than that of patients with colorectal cancer^3^; the one‐year survival rate is 58%‐76%, and the median survival time for patients with liver metastasis is 7.5 months.^4^, ^6^, ^7^ Therefore, confirming a diagnosis of MANEC is important. NETs have histologically characteristic features such as rosette growth patterns and a salt and pepper appearance. Although NETs per se are easy to diagnose, MANECs are difficult to diagnosis because of the similarity between NEC and poorly differentiated adenocarcinoma components. Thus, immunohistochemistry is recommended in these situations.

In general, highly malignant MANECs are difficult to treat; according to the National Comprehensive Cancer Network (NCCN) guidelines,^8^ the only curative option is surgical resection. In contrast, MANECs usually require more complex management such as chemotherapy for unresectable tumors or postoperative adjuvant therapy. The NCCN recommends either carboplatin +etoposide or cisplatin +irinotecan, which are derived from regimens used for treating small‐cell lung carcinoma.^8^ However, the most effective regimen for MANEC remains unclear because of the small number of relevant studies performed. In fact, although the response rates of patients with pulmonary small‐cell carcinoma to the aforementioned regimens are good (68% and 84%, respectively), they do not appear to prolong survival.^9^ The JCOG 1213 (UMIN000014795) and NECTOR (UMIN000012752) trials of these agents in patients with NEC are ongoing in Japan.

According to recent reviews, most endocrine cell carcinomas of the colon and rectum are considered MANECs^7^, ^10^; as ≥30% of these tumors’ components comprise adenocarcinoma, chemotherapy intended to target colorectal cancer may be suitable against MANECs. Moreover, a recent study^11^ showed that the KRAS status on the identical locus was common to both tumor components of their MANEC patients. In our case, we did not examine the KRAS pattern of the metastatic tumors; however, the lower tumor shrinkage rate after withdrawing panitumumab suggests that panitumumab could be effective for MANEC.

Regarding the cellular origins of MANECs, NEC shares a genetic profile with conventional colorectal adenocarcinoma. Hence, these components appear to be derived from the same cellular origin as amphicrine tumor, that is, most likely pluripotent epithelial stem cells or an adenocarcinoma precursor cells that are triggered by selective cytokines in the tumor environment or by somatic mutations.^12^, ^13^, ^14^ Therefore, MANECs may respond to chemotherapeutic agents that target colorectal adenocarcinoma.^15^ Apostolidis et al^16^ reported that as first‐line therapy, there was no significant difference between platinum and etoposide, or oxaliplatin and 5‐fluorouracil‐based regimens.

Our patient's diagnosis was corrected to reflect MANEC of the colon with liver metastasis following postoperative histopathological examination. Initially, the liver metastasis was judged to be unresectable because it invaded the umbilical portion, and thus, FOLFOX +panitumumab was administered. However, the resulting marked tumor regression made R0 resection possible. Moreover, considering that this patient survived 35 months after initial surgery, which surpassed the reported postoperative median survival time of patients with such tumors.^4^, ^6^, ^7^ Chemotherapy of colonic adenocarcinoma may also be effective against colon MANECs. In this case, because of the areas where positive for both NET marker and CEA, a clear proportion of both components are unknown. However, based on morphological and immunohistochemical findings, these tumors were assumed to amphicrine tumors, and thus, a regimen for adenocarcinoma was considered to have been effective for both components. The benefit of chemotherapy ought to be evaluated based on the characteristics and quantitative ratios of the components. However, circumspection is required for interpreting these results because the observation of the ratios of component is obtained from only a small portion of the whole tumor in the pathological specimen. Indeed, not many MANECs are diagnosed based on preoperative biopsy,^17^ and NECs sometimes exhibit tissue patterns similar to poorly differentiated adenocarcinoma and undifferentiated carcinoma on hematoxylin and eosin staining, making it difficult to differentiate between the tumor types. Hence, actual MANEC may present in patients diagnosed with poorly differentiated adenocarcinoma, as in our case. Therefore, it is important to perform detailed immunological examinations during differential diagnoses and to consider the possibility of MANEC.

In terms of the surgical indication and appropriate timing to perform operation for initially unresectable cases, several reports on colorectal cancers have revealed that after conversion surgery, the cases had an equally good prognosis as initially resectable cases.^18^, ^19^, ^20^ Furthermore, performing surgery at an appropriate time is recommended when curative resection is possible following chemotherapy.^21^, ^22^ Besides our case, no other reports of conversion surgery for liver metastases arising from colorectal MANEC have been previously reported, and its usefulness is therefore unknown. However, there are reports^23^, ^24^ of extending survival by performing R0 resection following mass reduction using preoperative chemotherapy, although they are cases of MANEC of the gallbladder. This suggests that conversion surgery is useful. In our case, we prepared for surgery immediately after deeming the lesions resectable, and the postoperative survival time was superior to that in several other reported patients with MANEC.^4^, ^6^, ^7^ Thus, we conjecture that the adaptation and timing of conversion surgery were appropriate. We anticipate that additional investigations will help determine optimal treatment modalities for patients with MANEC.

## 4 CONCLUSIONS

MANEC of the colon generally has a very poor prognosis; therefore, multidisciplinary treatment, including surgery, is important. Although the benefit of adjuvant chemotherapy remains unclear, regimens used for colorectal cancer may be effective for MANECs of the colon. Careful histopathological examination is important for the correct diagnosis of challenging cases.

## CONFLICT OF INTEREST

None declared.

## AUTHOR CONTRIBUTIONS

OS: conceived the study, participated in its design and coordination, collected the data, and prepared the draft of the manuscript. DS: evaluated pathological data. TM: evaluated pathological data.TT: conceived the study, participated in its design and coordination, and helped draft the manuscript. All authors read and approved the final manuscript.

Consent: Written informed consent for publication of the clinical details was obtained from the patient while his alive.
